# The Diagnosis Felt(y) Right: A Case Report of Felty Syndrome With Limited Articular Involvement

**DOI:** 10.7759/cureus.24593

**Published:** 2022-04-29

**Authors:** Victor E Serrano Santiago, Zack Morgan

**Affiliations:** 1 Internal Medicine, Methodist Health System, Dallas, USA

**Keywords:** anti-ccp, rheumatoid factor, bone marrow biopsy, pancytopenia, neutropenia, rheumatoid arthritis, splenomegaly, felty’s syndrome

## Abstract

We present a case of a 56-year-old female patient who presented to the emergency department with a one-day history of fever and confusion. She was found to have splenomegaly, multiple swan neck deformities, and pancytopenia. Chart review revealed that she had a three-year history of pancytopenia with two prior non-diagnostic bone marrow biopsies. Rheumatoid factor and cyclic citrullinated peptide antibody levels were elevated. The patient was ultimately diagnosed with Felty’s syndrome (FS). Felty’s syndrome is characterized by neutropenia, splenomegaly, and rheumatoid arthritis. This disease usually presents years after a diagnosis of rheumatoid arthritis is made. The neutropenia associated with Felty's syndrome can lead to life-threatening infections and therefore must be recognized so that the underlying cause of immunosuppression can be addressed.

## Introduction

Felty’s syndrome (FS) is characterized by the triad of neutropenia, splenomegaly, and rheumatoid arthritis. It typically presents in female patients who have had rheumatoid arthritis for at least 20 years. Here, we present a case of Felty's syndrome that presented with altered mentation without any known history of rheumatoid arthritis.

## Case presentation

A 56-year-old female presented to the emergency department with a one-day history of fever and confusion. She has a past medical history of idiopathic pancytopenia, present two years prior to admission, hypertension, and diabetes mellitus type 2. The initial work-up with a CT of the head was without any acute abnormality. A CTA of the head and neck was only notable for a small cerebellar AV malformation. Initial lab work was notable for a leukopenia of 2.1 × 10^3^/µL, with a neutrophil percentage of 9.1% (ANC: 191), hemoglobin of 5.3 g/dL, and platelets of 120 × 10^3^/µL. The physical exam was notable for splenomegaly. The family history was notable for a history of lupus in the patient’s mother. The patient’s prior pancytopenia workup had included two prior bone marrow biopsies, the first in February 2018, notable for "reactive bone marrow," and the second biopsy in November 2019, which was negative for abnormal cell populations. On both occasions, the patient was treated with a granulocyte colony-stimulating factor.

**Figure 1:**
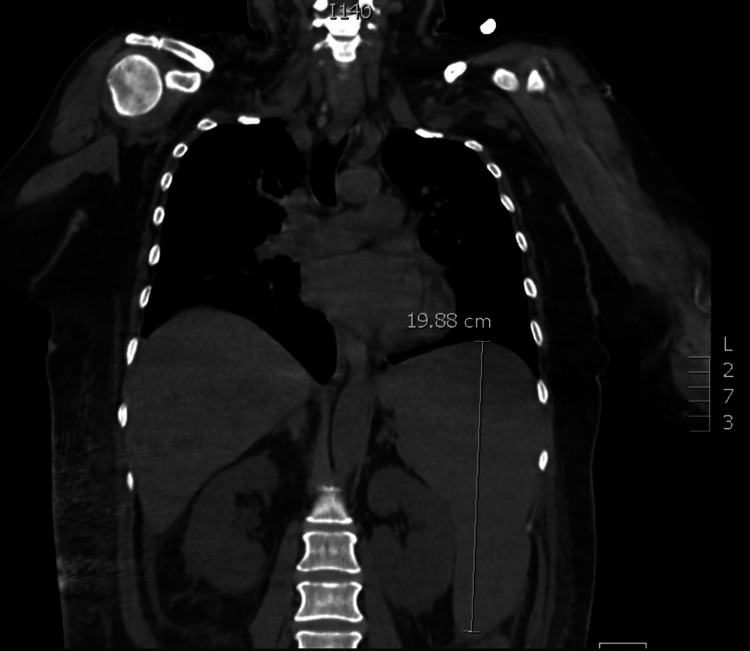
CT of the chest/abdomen showing severe splenomegaly

Her confusion improved, and her mental status returned to baseline, but her fevers persisted. Antibiotic treatment for neutropenic fever was started, and antinuclear antibodies and extractable nuclear antigens were sent for consideration of autoimmune etiologies. Given the continuing fevers in the setting of severe leukopenia, ID was consulted. The rheumatoid factor was notable at 190.9 IU/mL, an antinuclear antibody (ANA) titer of 1:640, and a homogeneous pattern with an anti-extractable nuclear antigen (anti-ENA) panel being negative. Additional laboratory work obtained throughout the hospital stay included complement levels, notable for a low C3 and normal C4. The hepatitis panel, HIV, and ADAMTS13 (a disintegrin and metalloproteinase with a thrombospondin type 1 motif, member 13) were all negative. Creatinine up trended as well, with accompanying proteinuria and a protein to creatinine ratio of 45, prompting a nephrology consult. Kappa/lambda free light chains were elevated, but urine protein electrophoresis and serum protein electrophoresis were negative. The patient was started on intermittent hemodialysis for acute renal failure that was not improving. Given the persistent neutropenia, the oncology service was consulted for consideration of a bone marrow biopsy. A CT scan of the chest, abdomen, and pelvis was ordered on hospital day 6 and revealed severe splenomegaly, with the spleen measuring 19.88 cm (Figure 1) in the craniocaudal axis.

Cytomegalovirus, Epstein-Barr virus, human herpesvirus 6, and Parvovirus PCRs were given splenomegaly and returned negative. Peripheral flow cytometry showed an atypical T-cell population of 6%, consistent with a reactive T-cell population. Differential diagnoses at this time included T-cell lymphoma and Felty syndrome, with neutropenia, splenomegaly, and positive rheumatoid factor. A bone marrow biopsy showed marrow hypercellularity with peripheral pancytopenia. An MDS panel showed no abnormalities suggestive of MDS. Bilateral hand films were ordered to assess for evidence of rheumatoid arthritis given the lack of overt disease history. No bony erosions or joint abnormalities were seen. However, upon focused interview, the patient reported a self-limited history of inflammatory joint symptoms that occurred around two to three years prior to admission. Renal biopsy was pursued given persistent kidney failure and was notable for acute tubular necrosis and mild interstitial fibrosis with tubular atrophy. The patient was administered G-CSF during the hospital stay, and her ANC responded adequately. Her fevers subsided, and broad-spectrum antibiotics were discontinued. The patient experienced renal recovery and was taken off intermittent hemodialysis. The general surgery service was consulted for consideration of splenectomy, which was deferred to an outpatient basis. She was given a series of vaccinations against encapsulated organisms, giving her functional asplenia. The patient was discharged with rheumatology and general surgery follow-up.

## Discussion

FS is characterized by the triad of neutropenia with an absolute neutrophil count below 2000/µL, splenomegaly, and rheumatoid arthritis. It typically manifests in female patients who have had rheumatoid arthritis for at least 20 years [1]. FS develops in approximately 1% of patients with rheumatoid arthritis. The most common presentation of FS is with infections secondary to neutropenia. Severe infectious complications are also the largest contributor to increased mortality [1]. The neutropenia in FS is multifactorial, and explanations include splenomegaly and immunologic factors. Splenomegaly contributes to neutropenia via sequestration and peripheral destruction of neutrophils [2]. The indirect decrease of neutrophils via the decrease of circulating G-CSF has also been described. IgG antibodies against G-CSF have been found in close to 73% of patients [1,3]. It is important to rule out large granular lymphocytic (LGL) leukemia in patients with suspected FS, as the two have similar clinical appearances. Additionally, FS and LGL leukemia share a common human leukocyte antigen-DR4 (HLA-DR4); however, LGL leukemia demonstrates T-lymphocytes with CD 2, 3, 8, 16, and 57 positivity [1,4]. These mutations were not present in our patient, effectively differentiating these two entities. The diagnosis of FS is one that is made clinically. If FS is suspected, a complete blood count with differential, absolute neutrophil count, and peripheral smear should be ordered initially. An oncology consult may be warranted if abnormal cell populations are seen in a bone marrow biopsy to rule out other secondary causes of pancytopenia.

FS is highly associated with rheumatoid factor and cyclic citrullinated peptide antibodies, so these levels should be pursued [1]. It is important to be able to identify FS in patients without articular involvement or prior diagnosis of RA, as pancytopenia can be the presenting finding of RA [5]. Although rare, several cases of FS without articular RA have been presented in the literature [6]. Our patient did not have an established diagnosis of rheumatoid arthritis, but she did have swan-neck deformities on physical exam, suggestive of articular joint damage, and reported a history of albeit brief symptoms compatible with inflammatory arthritis. Our patient initially presented with neutropenic fever and pancytopenia, and was ultimately diagnosed with Felty’s syndrome based on splenomegaly, neutropenia, and serologic findings of seropositive rheumatoid arthritis. Treatment of FS is centered on resolving any neutropenia by addressing the underlying cause of rheumatoid arthritis and typically involves treatment with disease-modifying antirheumatic drugs. Granulocyte colony-stimulating factor is commonly used for the treatment of neutropenia until absolute neutrophil counts improve or in cases of systemic infection with neutropenia.

It is considered that for many years, splenectomy was the main therapy for SF. However, this surgical modality has now been largely superseded by medical therapy. The indications for splenectomy are now more limited [2], and it is not commonplace in management. Table 1 shows a comparison of multiple cases, including several cases from a prior case review [7] found in the literature, in which only 20% of patients underwent a splenectomy. Most patients in this review responded to disease-modifying antirheumatic drugs. Splenectomy should be pursued in the setting of severe neutropenia or thrombocytopenia, as splenectomies have been shown to improve peripheral white blood cell counts [8], though prophylactic splenectomies are not recommended [9]. Splenomegaly is secondary to the underlying pathogenesis of Felty’s syndrome [10], and as such, splenectomy provides a temporary improvement in the neutrophil count but is not recommended as a first-line, long-term solution for neutropenia. The mainstay of therapy for neutropenia continues to be disease-modifying anti-rheumatic drugs.

**Table 1:** Cases of Felty's syndrome Gender: M: male, F: female, NR: not reported, SP: seropositive disease with no specified antibodies, DMARD: disease-modifying antirheumatic drugs

Number	References	Age	Gender	Known RA	RF/CCP	Treatment	Splenectomy
1	Cornwell and Zacharski [11]	56	M	+	+/NR	Cyclophosphamide	No
2	Heyn [12], case #1	76	F	+	+/NR	Lithium carbonate	No
3	Heyn [12], case #2	55	M	+	+/NR	NA	No
4	Armstrong et al. [13], case #1	73	F	+	+/NR	NA	No
5	Armstrong et al. [13], case #2	57	F	+	+/NR	NA	No
6	Armstrong et al. [13], case #3	72	F	+	+/NR	NA	No
7	Bradley and Pinals [14]	66	F	+	+/NR	NA	Yes
8	Fitzgerald et al. [15]	58	M	+	+/NR	NA	Yes
9	Cycowitz et al. [16]	34	F	+	+/NR	Prednisone	No
10	Wagner et al. [17]	56	F	+	SP	DMARD	Yes
11	Talip et al. [18]	58	M	+	SP	Gold, prednisone, DMARD	No
12	Zakzook et al. [19]	30	F	+	NR	DMARD, plasmapheresis, prednisone	No
13	Ghavami et al. [20]	75	F	+	SP	Prednisone, etanercept, DMARDs	No
14	Shipley et al. [21]	74	F	+	+/+	Infliximab, rituximab	No
15	Chandra et al. [22]	60	M	+	SP	Prednisone, auranofin, DMARD, penicillamine, colchicine, infliximab, rituximab	No
16	Chavalitdhamrong et al. [23]	31	M	+	+/+	DMARD	No
17	Muroi et al. [24]	52	F	+	+/+	NA	Yes
18	Rozin et al. [25]	57	M	+	+/+	Prednisone, DMARD	No
19	Jain et al. [6]	73	F	+	+/+	Prednisone	No
20	Lagrutta et al. [26]	56	F	+	+/+	Prednisone, colchicine, DMARDs	Yes
21	Puksic et al. [27]	54	F	+	SP	Gold, DMARD, rituximab	No
22	Hamada-Ode et al. [28]	74	F	+	NR	DMARD, methylprednisolone, abatacept	No
23	Liatsos et al. [29]	73	F	+	+/+	DMARD, methylprednisolone	No
24	Aslam et al. [7]	47	F	−	−/+	DMARD	No
25	Wang et al. [30]	66	M	+	NR	DMARD	No
26	Nimri et al. [31]	64	F	+	+/+	Prednisone, DMARD	No
27	Yang et al. [32]	48	F	+	+/ NR	Prednisone, DMARD	No
28	Wu et al. [33]	48	F	−	+/+	DMARD	No
29	Our case	56	F	−	+/+	DMARD	Yes

## Conclusions

Felty’s syndrome is diagnosed clinically with the triad of neutropenia, splenomegaly, and RA. FS should be considered in all patients presenting with any symptom of the classic triad, particularly if there are physical findings to suggest a history of inflammatory arthritis. It is vital to understand the risk of neutropenia given its role in increasing the mortality of FS. Early serologic workup and consideration of bone marrow aspiration should be pursued as it can support the diagnosis and differentiate it from LGL leukemia. DMARDs should be a first-line treatment, with splenectomy reserved for refractory cases or when prompt improvement in neutropenia is warranted.
